# Lipidomics of oxidized polyunsaturated fatty acids

**DOI:** 10.1016/j.freeradbiomed.2012.08.565

**Published:** 2013-06

**Authors:** Karen A. Massey, Anna Nicolaou

**Affiliations:** School of Pharmacy and Centre for Skin Sciences, School of Life Sciences, University of Bradford, Bradford BD7 1DP, UK

**Keywords:** Cyclooxygenase, Lipoxygenase, Cytochrome P450, Tandem mass spectrometry, Bioactive lipids, Eicosanoids, Docosanoids, Octadecanoids, Free radicals

## Abstract

Lipid mediators are produced from the oxidation of polyunsaturated fatty acids through enzymatic and free radical-mediated reactions. When subject to oxygenation via cyclooxygenases, lipoxygenases, and cytochrome P450 monooxygenases, polyunsaturated fatty acids give rise to an array of metabolites including eicosanoids, docosanoids, and octadecanoids. These potent bioactive lipids are involved in many biochemical and signaling pathways, with inflammation being of particular importance. Moreover, because they are produced by more than one pathway and substrate, and are present in a variety of biological milieus, their analysis is not always possible with conventional assays. Liquid chromatography coupled to electrospray mass spectrometry offers a versatile and sensitive approach for the analysis of bioactive lipids, allowing specific and accurate quantitation of multiple species present in the same sample. Here we explain the principles of this approach to mediator lipidomics and present detailed protocols for the assay of enzymatically produced oxygenated metabolites of polyunsaturated fatty acids that can be tailored to answer biological questions or facilitate assessment of nutritional and pharmacological interventions.

## Graphical abstract

**Graphical abstract:**

**Figure:**
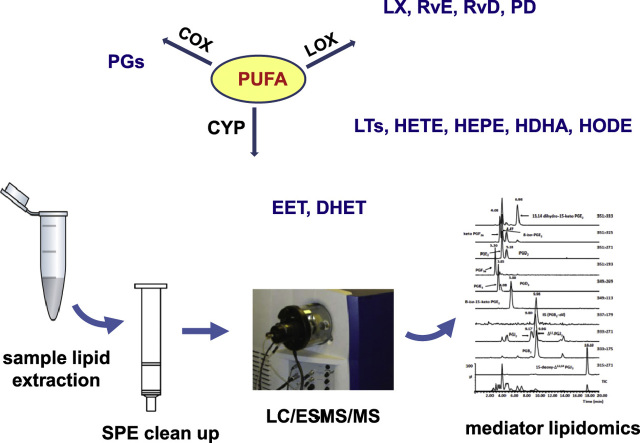

**Highlights:**

► Polyunsaturated fatty acids oxygenate to eicosanoids, docosanoids, and octadecanoids. ► We performed simultaneous analysis of lipids by liquid chromatography–tandem mass spectrometry. ► Multiple lipid mediators were detected at low picograms per milliliter and in various biological matrices. ► Mediator lipidomics are relevant to biological, nutritional, and pharmacological studies.

## Introduction

**Fig. 1:**
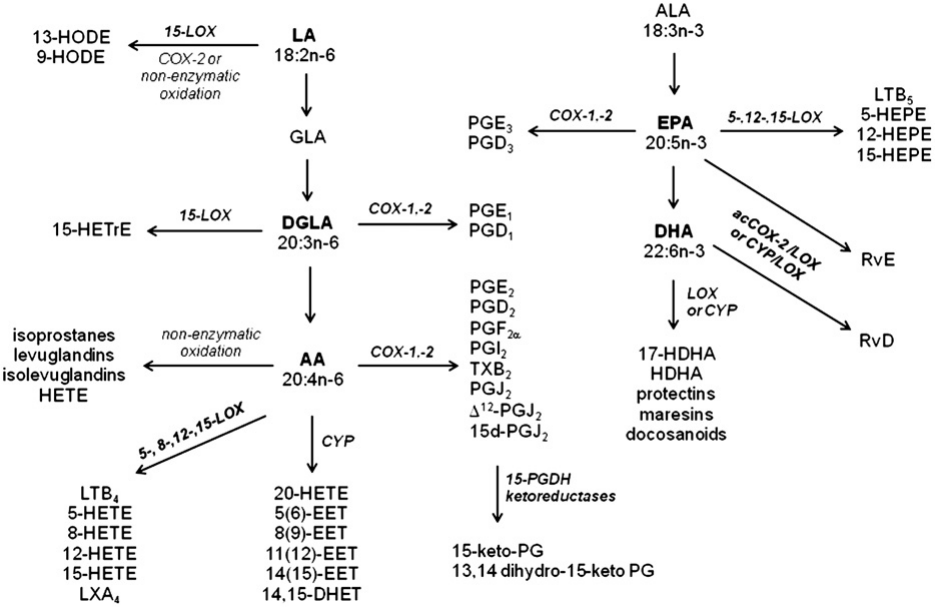
Schematic showing an abridged overview of the main pathways involved in the production of polyunsaturated fatty acid-derived oxygenated mediators. acCOX-2, acetylated COX-2.

Polyunsaturated fatty acids (PUFAs) are precursors of numerous metabolites endowed with potent bioactivities and involved in homeostatic and pathophysiological events in the majority of mammalian cells [1–4]. Although these lipid mediators are, primarily, products of enzyme-mediated oxygenations, compounds with similar structures can be produced by reactive oxygen species (ROS)-initiated reactions [5–8]. Enzymatically derived oxygenated PUFAs originate from three main pathways: the cyclooxygenase (COX), lipoxygenase (LOX), and cytochrome P450 (CYP) reactions (Fig. 1). The diversity of PUFA precursors that can be metabolized in this way is quite broad, resulting in a large number of mediators whose range of bioactivities continue to be the focus of current investigations, while new species are constantly being discovered and their activities remain to be explored.

One of the most studied families of bioactive lipids are the eicosanoids. These C20 carboxylic acids are products of arachidonic acid (AA; 20:4*n*−6), eicosapentaenoic acid (EPA; 20:5*n*−3), and dihomo-γ-linolenic acid (DGLA; 20:3*n*−6) (Fig. 1). Best known for their pivotal role in inflammation, AA-derived eicosanoids are considered to be predominantly proinflammatory, whereas EPA- and DGLA-derived mediators are believed to either oppose or dampen down this effect, altering the biological milieu to an anti-inflammatory one [9]. When metabolized by COX isoforms (i.e., the constitutive COX-1 or inducible COX-2) and subject to the prevalence of terminal synthases these C20-PUFA generate prostaglandins (PGs), prostacyclin, and thromboxanes (TXs), collectively termed *prostanoids* [10,11]. Catabolism through dehydrogenation and reduction results in the formation of metabolites with significantly reduced bioactivities (i.e., 15-keto- and 13,14-dihydro-15-keto PGs), representing part of the biological mechanism that controls tissue levels of bioactive lipids [12].

LOX enzymes insert OH groups in a stereoselective manner and on a variety of PUFA substrates, including esterified acyl species [13–15]. However, LOX activities are commonly defined by their positional selectivity when they oxygenate AA: in this way the main mammalian LOX enzymes are defined as 5-LOX, 8-LOX, 12-LOX, and 15-LOX. With the exception of the unique to mammalian skin 12*R*-LOX, all other LOX isoforms give hydroxy fatty acids of the *S* configuration. LOX isozymes initially produce unstable hydroperoxides that are then reduced to hydroxy acids [15]. When subjected to LOX oxygenation PUFAs can generate an array of mono- and polyhydroxy fatty acids: e.g., AA produces hydroxyeicosatetraenoic acids (HETEs), leukotrienes (LTs), and lipoxins (LXs); EPA generates hydroxyeicosapentaenoic acids (HEPEs) and E-series resolvins (RvE's); docosahexaenoic acid (DHA; 22:6*n*−3) produces docosanoids including hydroxydocosahexaenoic acids (HDHAs), maresins, D-series resolvins (RvD's), and protectins (PDs); linoleic acid (LA; 18:2*n*−6) forms octadecanoids such as hydroxyoctadecadienoic acids (HODEs); DGLA forms hydroxyeicosatrienoic acids (HETrE's), etc. (Fig. 1). Interestingly, partially inhibited COX-2 (e.g., acetylated COX-2 after treatment with aspirin) can generate LOX-like products with the OH group at the *R* configuration, e.g., 15*(R)*-HETE from AA and 18(*R*)-HEPE from EPA. These products are important metabolic precursors of 15-*epi*-LXA_4_ and RvE_1_, respectively, which, in turn, are generated through sequential LOX reactions via transcellular metabolism [16,17].

CYP monooxygenases relevant to PUFA metabolism catalyze epoxygenations and midchain and ω-hydroxylations [18–20]. Substrate specificity and stereoselectivity varies widely between CYP isoforms, e.g., when using AA as substrate CYP can form epoxides on every one of the four double bonds of the acyl chain, forming epoxyeicosatrienoic acid (EET) regioisomers that are further metabolized by epoxide hydrolases to biologically inactive dihydroeicosatetraenoic acids (DHET) (Fig. 1). Midchain hydroxylations produce a range of LOX-like hydroxy fatty acids (e.g., HETE, HEPE) although these mediators are not necessarily of the *S* configuration and may not even be enantiomerically pure but racemic mixtures (reviewed in [5]).

Finally, ROS-mediated reactions can form a range of small lipid molecules with structures resembling these of enzymatically produced mediators (Fig. 1). Examples include the isoprostanes, a family of PG-like regio- and stereoisomeric derivatives formed through the oxidation of phospholipid esterified PUFAs, the highly reactive keto-aldehydes levuglandins and isolevuglandins, as well as a wide range of monohydroxy fatty acids formed as racemic mixtures [6,8,21–23].

Given the immense biological importance, increasing number, and diversity of PUFA-derived oxygenated metabolites, there is a clear need for a sensitive, selective, and accurate assay system suitable for the qualitative and quantitative analysis of these lipid species. Currently, analysis of eicosanoids and other oxygenated PUFA mediators can be performed using various methodologies: enzyme-linked immunosorbent assays and radioimmunoassays are popular but can measure only one metabolite at a time, are not always selective, can be subject to cross-reactivity, and are available only for certain lipids [24,25]. Gas chromatography coupled to mass spectrometry (GC–MS) or tandem mass spectrometry (GC–MS/MS) has been successfully applied to eicosanoid research, although the need to derivatize the lipids to form volatile species causes limitations including the danger of thermal decomposition [26–28]. High-pressure liquid chromatography (HPLC) with fluorescence detection requires derivatization, whereas HPLC–UV is lacking sensitivity and is applicable only to a limited number of UV-active mediators [29–31]. However, the versatility and high separation power of liquid chromatography (LC—as HPLC or UPLC) when coupled to tandem mass spectrometry (LC–MS/MS) have been proven to be an excellent analytical platform for mediator lipidomic assays with detection limits in the picogram range [32–37].

Overall, mass spectrometry-based mediator lipidomics offers a diverse dynamic tool for the simultaneous analysis of multiple mediators formed by various biochemical routes and all present in one single sample and has already made its mark on lipid research: the approach has facilitated the discovery of novel lipid species while being successfully applied to diverse biological matrices including plasma, brain, liver, pancreas, cutaneous blister fluid, myometrial tissue, spinal fluid, breath condensate, cell culture media, solid tumors, urine, etc. [33–35,38–47]. In this article we describe detailed experimental protocols for the study of enzymatically produced oxygenated metabolites of PUFAs including their extraction from various biological materials, quantitation, and elucidation of chirality. These protocols can be tailored to answer targeted or untargeted research questions, investigate the origin of species of interest, and assess effectiveness of nutritional and therapeutic interventions.

## Principles

**Fig. 2:**
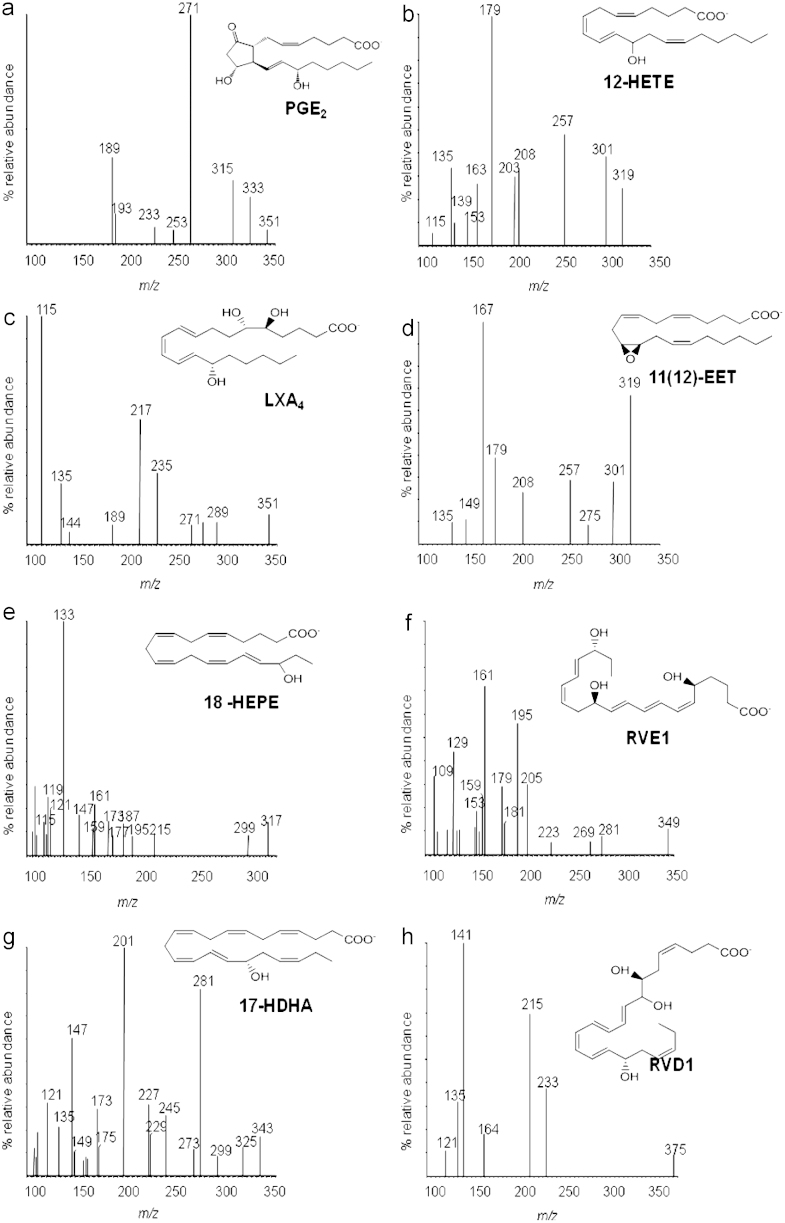
ESI–MS spectra for the AA-derived mediators PGE_2_, 12-HETE, LXA_4_, and 11(12)-EET, the EPA-derived 18-HEPE and RvE_1_, and the DHA-derived 17-HDHA and RvD_1_.

Mass spectrometry measures the mass-to charge (*m*/*z*) ratio of ionized molecules. Further fragmentation of molecular ion species generates product ions that provide structural information for the compound of interest and can inform the development of sensitive quantitative assays. Electrospray ionization (ESI) is a low-energy (soft) ionization technique applicable to the qualitative and quantitative analysis of lipid species [48,49]. Although ESI generates both positively and negatively ionized species, most applications relevant to oxygenated PUFA mediators are in the negative-ion mode (ES^−^), in which they form [M-H]^−^ carboxylate ion species in high abundance [50] (Fig. 2).

Tandem mass spectrometry involves multiple rounds of mass spectrometry in which one mass analyzer produces compound-specific fragment ions that can then be selected and further fragmented, with the resulting product ions studied through a second mass analyzer. This setting allows a number of experimental routines such as the widely used multiple-reaction monitoring (MRM) mode. In a typical MRM experiment the ion of interest is isolated in the first mass analyzer (quadrupole analyzer) and fragmented in a collision cell using gas such as argon, and the product ions are then scanned and selected by a second analyzer (e.g., a second quadrupole) and related back to their precursor ion. In this way an MRM transition monitors the formation of compound-specific fragments giving a very selective and highly sensitive assay system, with much reduced background noise, and is immensely useful for quantitative approaches [51].

Oxygenated PUFA mediators are predominantly hydroxylated unsaturated carboxylic acids that are typically separated by polarity using, mostly, C18 reverse-phase columns and acidified methanol or acetonitrile-based mobile phase systems [33,34]. One of the advantages of ESI is that it can be readily coupled to HPLC, offering a great advantage for the development of LC/ESI–MS/MS assays. MRM assays allow the simultaneous detection of multiple lipid species using analyte-specific information deduced from their MS/MS fragmentation patterns. The dwell time and intrascan delay determine how many such assays can be monitored in each run (typically 20–30 analytes depending on instrument specifications) although the MRM protocol can be segmented during the run to allow for a much larger number of mediators to be assessed.

Furthermore, LC/ESI–MS/MS allows the analysis of coeluting compounds that can be monitored via different MRM transitions, whereas distinction of isobaric species is achieved by chromatographic separation [34]. Although some diastereomers could be separated by reverse-phase columns, separation of enantiomeric species requires the use of chiral columns. Amylose or cellulose stationary phases that can be run with solvents compatible with ESI offer good sensitivity [52] (Fig. 4). Finally, quantitation is based on the use of deuterated internal standards and calibration lines constructed using commercially available or custom-made synthetic standards.

Although PGs and hydroxy and epoxy fatty acids can be simultaneously analyzed [36,37], here we present two separate assays for the COX- and LOX/CYP-derived mediators. We wanted the method to include series 1, 2, and 3 prostanoids (DGLA, AA, and EPA products) as well as deactivated 15-keto- and 13,14-dihydro keto-PG derivatives, so that it could be applied to various clinical and nutritional studies, including interventions with *n*−3/*n*−6 PUFAs. We observed that when we combined both protocols (COX and LOX/CYP) the resulting LC–MS/MS chromatogram was very crowded, with most mediators eluting during the early part of the run. Many of these mediators share common fragment ions, whereas others, such as PGE/PGD, are isobaric species, and therefore good chromatographic separation became necessary for accurate quantitation, especially when analyzing lipids from various biological matrices. To achieve optimal separation of isobaric species we used an acetonitrile-based solvent system. However, hydroxy fatty acids are less polar than prostanoids and interact more strongly with a C18 column, leading to peak broadening; hence a more polar solvent such as methanol was required to give sharper peaks and improve resolution [30]. Resolution of hydroxy fatty acids was further improved when a core shell (pore size 2.6 μm) column was employed; however, this type of column did not improve the chromatography of prostanoids.

## Materials

Solid-phase extraction cartridges STRATA SPE cartridges, C18-E (500 mg, 6 ml; Cat. No. 8B-S001-HCH), amber glass vials with screw cap (2.0 ml; Cat. No. ARO-3811), conical glass inserts (100 μl; Cat. No. ARO-4520), open-top screw caps (Cat. No. ARO-8897), and PTFE septa (8-mm diameter, 0.010 in.; Cat. No. ARO-6817) were from Phenomenex (Macclesfield, UK). Ten-microliter fixed-needle gas-tight glass syringe (Cat. No. SZR-830-190R), 50-μl fixed-needle gas-tight glass syringe (Cat. No. SZR-830-210X), 250-μl fixed-needle gas-tight glass syringe (Cat. No. SZR-830-270T), pH indicator paper sticks, nonbleeding, supplied with color comparison chart pH 1.7 to 3.8 (85×6 mm; Cat. No. FB33007), borosilicate glass round-bottomed screw-neck tubes (16×100 mm; Cat. No. TKV-173-020E) with screw caps (PTFE liner; Cat. No. TKV-178-020U), glass Pasteur pipettes, and all other lab glassware were from Fisher Scientific (Loughborough, UK). Plain glass wide-neck vials, capped (10 ml; Cat. No. 116105), were from Laboratory Sales Ltd (Rochdale, UK). Nitrogen (oxygen-free) (Cat. No. UN1066) was from BOC (Manchester, UK). Glacial acetic acid (ReagentPlus; Cat. No. A6283), hydrochloric acid (HCl; ACS reagent; Cat. No. 320331), and sodium hydroxide (NaOH; ACS reagent; Cat. No. 221465) were from Sigma–Aldrich Co. Ltd (Dorset, UK). Methanol (Cat. No. M/4056/17), acetonitrile (Cat. No. A/0626/17), ethanol (Cat. No. E/0665DF/17), hexane (Cat. No. H/0406/17), and methyl formate (Cat. No. 12682-0025) were all of HPLC-grade and purchased from Fisher Scientific. Deionized water was from an ELGA system (18.2 MΩ-cm purity, Model Ultra Ionic, Part No. PRIPLB0450, High Wycombe, UK). RC DC protein assay kit II using bovine serum albumin (BSA) as protein standard (Cat. No. 500-0122) was from Bio-Rad Laboratories Ltd (Hemel Hempstead, UK). Ninety-six-well microplates (PS, sterile; Cat. No. 655161) were from Greiner Bio-One Ltd (Stonehouse, UK).

The following analytical-grade standards were purchased from Cayman Chemical Co. (Ann Arbor, MI, USA): 15-deoxy-Δ^12,14^-PGJ_2_ (Cat. No. 18570), PGJ_2_ (Cat. No. 18500), Δ^12^-PGJ_2_ (Cat. No. 18550), 15-keto-PGE_2_ (Cat. No. 14720), PGE_3_ (Cat. No. 14990), PGD_3_ (Cat. No. 12990), PGE_2_ (Cat. No. 14010), PGD_2_ (Cat. No. 12010), PGF_2α_ (Cat. No. 16010), PGE_1_ (Cat. No. 13010), PGD_1_ (Cat. No. 12000), PGF_1α_ (Cat. No. 15010), TXB_3_ (Cat. No. 19990), TXB_2_ (Cat. No. 19030), 6-keto-PGF_1α_ (Cat. No. 15210), 13,14-dihydro-PGF_2α_ (Cat. No. 16660), 13,14-dihydro-PGE_1_ (Cat. No. 13610), 13,14-dihydro-15-keto-PGE_2_ (Cat. No. 14650), 13,14-dihydro-15-keto-PGF_2α_ (Cat. No. 16670), 13,14-dihydro-15-keto-PGE_1_ (Cat. No. 13650), 13,14-dihydro-15-keto-PGF_1α_ (Cat. No. 15670), PGB_2_-*d*_4_ (Cat. No. 311210), ±9-HODE (Cat. No. 38400), ±13-HODE (Cat. No. 38600), ±5-HEPE (Cat. No. 32200), ±8-HEPE (Cat. No. 32340), ±9-HEPE (Cat. No. 32400), ±11-HEPE (Cat. No. 32500), ±12-HEPE (Cat. No. 32540), ±15-HEPE (Cat. No. 32700), ±18-HEPE (Cat. No. 32840), ±5-HETE (Cat. No. 34210), ±8-HETE (Cat. No. 34340), ±9-HETE (Cat. No. 34400), ±11-HETE (Cat. No. 34500), ±12-HETE (Cat. No. 34550), ±15-HETE (Cat. No. 34700), ±10-HDHA (Cat. No. 33400; ±13-HDHA (Cat. No. 33500), ±14-HDHA (Cat. No. 33550), ±17-HDHA (Cat. No. 33650), ±20-HDHA (Cat. No. 33750), 15(*S*)-HETrE (Cat. No. 36720), LTB_4_ (Cat. No. 20110), RvD_1_ (Cat. No. 10012554), 5(*S*),6(*R*),15(*S*)-LXA_4_ (Cat. No. 90410), 5(*S*),6(*R*),15(*R*)-LXA_4_ (15-epi-LXA_4_; Cat. No. 90415), 5-oxo-ETE (Cat. No. 90415), ±5(6)-EET (Cat. No. 50211), ±8(9)-EET (Cat. No. 50351), ±11(12)-EET (Cat. No. 50511), ±14(15)-EET (Cat. No. 50651), ±5,6-DHET (Cat. No. 51211), ±8,9-DHET (Cat. No. 51351), ±11,12-DHET (Cat. No. 51511), ±14,15-DHET (Cat. No. 51651), and 12(*S*)-HETE-*d*_8_ (Cat. No. 334570). LTB_5_ (Cat. No. BML-LB005-0050) was from Enzo Life Sciences (Exeter, UK) and RvE_1_ (Cat. No. 10007848) was from Cayman Europe (Tallinn, Estonia). PD_1_ was kindly provided by Dr. C.N. Serhan, Harvard Medical School (Boston, MA, USA).

## Instrumentation

Solid tissue samples were homogenized using a Dounce tissue grinder with pestle (1 ml; Cat. No. FB56687) from Fisher Scientific or an Ystral homogenizer X10/25 (10-mm-diameter shaft, set at speed of 11 kHz) from The Scientific Instrument Centre Ltd (Winchester, UK). Solid-phase extraction (SPE) was performed using a 12-position vacuum manifold set (Cat. No. AHO-6023) and the solvents were removed using an SPE 12-position drying attachment with nitrogen supply (Cat. No. AHO-6050), all from Phenomenex. The analysis of lipid mediators was performed using the following HPLC columns: C18 Luna (150×2.0 mm, 5 μm, 100-Å particle size; Cat. No. 00F-4252-B0), C18 Kinetex (100×2.1 mm, 2.6 μm, 100-Å particle size; Cat. No. 00D-4462-AN), and LUX Cellulose-1 (150×2.0 mm, 3 μm, 1000-Å particle size; Cat. No. 00F-4458-B0), also from Phenomenex. All columns were fitted with guard columns filled with the same material: security guard cartridges C18 (4×2-mm i.d., Cat. No. AJO-4286) with security guard analytical cartridge holder (Cat. No. KJO-4282), KrudKatcher Ultra inline filter (0.5-μm depth filter×0.004-in. i.d., Cat. No. AFO-8497), security guard cartridges LUX cellulose-1 (4×2.0-mm i.d., Cat. No. AJO-8402) with security guard analytical cartridge holder (Cat. No. KJO-4282), also supplied by Phenomenex. LC/ESI–MS/MS analysis was performed on a HPLC pump (Alliance 2695; Waters, Elstree, UK) with autosampler (2690; Waters) coupled to a triple-quadrupole mass spectrometer with ESI probe (Quattro Ultima; Waters). Instrument handling and data management were done through MassLynx version 4.0 (Waters). Ninety-six-well microplates were read using a Labsystems Multiskan RC microplate reader version 6.0 (650 nm, Cat. No. 1506250) operated using Gemini 4.0 software (Product No. 5197051; Labsystems Oy, Helsinki, Finland). A Fisions Whirlimixer (Model WM/250/F; Fisions Science Apparatus, Loughborough, UK) and a refrigerated bench-top centrifuge (Model RT6000B; Sorvall, UK) were also used.

## Protocol

**Fig. 3:**
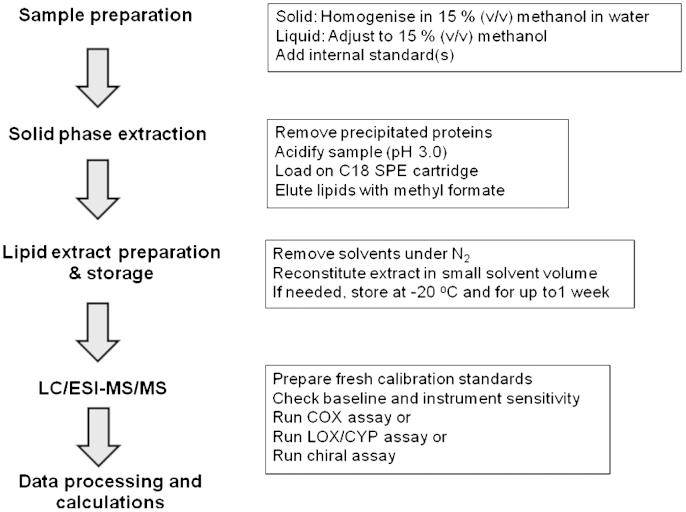
Flow chart showing the main steps for the LC/ESI–MS/MS mediator lipidomic assay.

The main steps and workflow of this protocol are shown in Fig. 3. Overall, solid samples are homogenized in 15% methanol/water (v/v) and liquid samples are adjusted to 15% methanol (v/v). After this initial step, the homogenate is spiked with the appropriate internal standards: PGB_2_-*d*_4_ for the COX-mediator assay and 12(*S*)-HETE-*d*_8_ for the LOX/CYP and chiral assays. The samples are then acidified to pH 3.0 and semipurified by SPE. The resulting lipid extract is analyzed by LC/ESI–MS/MS using the appropriate solvent system and column. Specific details are as follows.

### Sample preparation and homogenization

#### A. Preparation of liquid sample volume <200 μl

This procedure is typically applied to cutaneous blister fluid or other small-volume liquid samples. Defrost the sample on ice and in the dark. *Note. Do not use a water bath to defrost a sample; the sample can be warmed by using your hand to the point at which a small amount of ice remains, thus indicating that the sample temperature is still close to 0 *°*C.* If required, remove a suitable aliquot for protein content determination (10–20 μl) and store it separately. Transfer the defrosted liquid sample into a clean plain glass wide-neck vial (sample vial) while carefully measuring and recording the volume. Add 1 ml of ice-cold 15% methanol in water (v/v) and then wash the storage vial with 2×1 ml of cold 15% methanol in water (v/v) to collect any remaining biological sample. Transfer the washes to the sample vial using a glass Pasteur pipette; the final sample volume should be approximately 3 ml. Add 40 ng each of freshly prepared internal standards PGB_2_-*d*_4_ and 12(*S*)-HETE-*d*_8_ (40 μl of 1 ng/μl in ethanol). Place the lid on the sample vial and leave the sample homogenate on ice and in the dark for 15 min.

#### B. Preparation of liquid sample volume from 5 ml to 200 μl

This procedure is typically applied to preparation of plasma samples. Defrost the sample on ice and in the dark but do not use a water bath to defrost a sample (see Section A). If required, remove a suitable aliquot for protein content determination and transfer the liquid into a clean plain glass wide-neck vial (sample vial) while carefully measuring and recording the volume. Add an appropriate volume of ice-cold water to bring the solution to a final volume of 4 ml. Then add 700 μl of ice-cold methanol to bring the solution to 15% methanol (v/v). For sample volumes that are over 2 ml it is advisable to add ice-cold water up to a final volume of 6 ml (maximum SPE capacity) and adjust the volume of methanol accordingly. In all cases, add 40 ng of freshly prepared internal standards and continue with the protocol described in Section A.

#### C. Preparation of cell culture medium from 10 to 5 ml

Defrost the sample on ice and in the dark but do not use a water bath (see comments in Section A). Add an appropriate volume of ice-cold methanol directly to the sample while in its original container to bring it up to 15% methanol (v/v): i.e., for a 10-ml sample a volume of 1.77 ml ice-cold methanol would be needed. Then add 40 ng of freshly prepared internal standards and continue with the protocol described in Section A.

#### D. Preparation of solid tissue sample

*Note. For LC/ESI–MS/MS analysis a tissue sample should typically weigh between 45 and 200 mg. Extracts from samples >200 mg can potentially cause noticeable ion-suppression effects and can contaminate the LC–MS system. Therefore for tissue samples larger than 200 mg, it is necessary to cut an appropriately sized section before extraction*. For “soft” tissues (e.g., brain) place the frozen tissue directly into the Dounce tissue grinder and add 500 μl of ice-cold methanol. Keeping the tissue grinder on ice, break up the tissue using the pestle until fully homogenized. If required, remove an aliquot of the suspension for protein content analysis. Then, transfer the homogenate into a clean plain glass wide-neck vial (sample vial), using a glass Pasteur pipette. Wash the tissue grinder and pestle with 2×100 μl of ice-cold methanol and transfer the washes into the sample vial always using a glass Pasteur pipette. Add 4 ml of ice-cold water to the sample vial to bring the homogenate to 15% methanol in water (v/v). Then add 40 ng of freshly prepared internal standards and continue with the protocol described in Section A. *Note. Carefully wash the tissue grinder and pestle with analytical-grade water and ethanol in between samples to prevent cross-contamination*.

When dealing with “tough” tissues (e.g., muscle, skin sections) place the frozen tissue directly into a clean plain glass wide-neck vial (sample vial) containing 4 ml of ice-cold 15% methanol (v/v) in water. Homogenize the tissue using the Ystral homogenizer X10/25 in 4×15-s bursts always keeping the solution on ice in between. If required, remove an aliquot of the suspension for protein content analysis. Then add 40 ng of freshly prepared internal standards and continue with the protocol described in Section A. *Note. Carefully wash the blade homogenizer with analytical-grade water and ethanol in between samples to prevent cross-contamination.*

### Solid-phase extraction

After incubating the homogenate on ice, centrifuge it at 4 °C, 5000 rpm, for 10 min to remove any precipitated proteins. *Note. If necessary, retain the resulting pellet for protein content determination and store separately.* Transfer the clear supernatant to a clean plain glass wide-neck vial. Acidify each supernatant to pH 3 using 20–30 drops of 0.1 M HCl (0.1 M HCl: 850 μl concentrated HCl in 100 ml water); this should be sufficient for small-volume liquid and solid samples. For plasma samples and cell culture media use 1 M HCl (20–30 drops should be sufficient to bring the sample pH to 3.0). *Note. This step is necessary to fully protonate the lipid mediator species, allowing them to be retained on the C18 SPE cartridge. However, care must be taken to acidify the samples just before loading them to the preconditioned SPE cartridges.*

Before acidifying the samples, attach the required number of SPE cartridges to the vacuum manifold. Place a waste bucket in the manifold to collect and discard the washes. *Note. Wash volumes used in SPE are determined by the mass of the sorbent bed, typically 1* ml *of solvent is needed per 100* mg *of bed mass. However, when extracting biological material for LC/ESI–MS/MS analysis it is vital to remove all weakly bound impurities from the sorbent bed. If this is not done effectively, impurities in the final lipid mediator extract may cause ion-suppression effects, leading to a loss in mass spectrometer sensitivity (see Caveats). Therefore, larger wash volumes are used (4*×*5* ml *on 500* mg *sorbent bed) to ensure that the extract is as pure as possible before analysis.* Activate the appropriate number of cartridges by washing them with 20 ml of methanol followed by 20 of ml water. At the end of this step, close the taps to stop the flow. *Note. Do not allow the sorbent bed to run dry at any point during either the activation or the running of SPE.*

Transfer one of the acidified ice-cold samples onto one of the activated cartridges using a glass Pasteur pipette. Open the tap and allow the liquid to pass through in a drop-wise manner. Apply vacuum if necessary. However, do not allow the flow rate to become too fast as this will reduce the efficiency at which the lipid mediator species will be retained onto the cartridge. When all the sample has passed through the SPE and you can see only a few drops left at the top of the cartridge, close the tap to stop the sample from running through the SPE. Repeat this step for all the acidified samples. Once the samples have been loaded, wash the cartridges with 20 ml 15% (v/v) methanol in water, followed by 20 ml water to remove excess salts and other water-soluble impurities. Then wash with 10 ml hexane to remove any water. Stop the vacuum and replace the waste container with borosilicate glass round-bottomed screw-neck tubes to collect the lipid extract; these vials should be placed in the manifold collection rack. Then wash the SPE with 12 ml methyl formate to collect the lipids. These extracts are then dried under nitrogen and in the dark (to prevent photodegradation) until completely dry.

### Lipid extract reconstitution and storage

When planning to analyze lipid mediators using either the COX or the LOX/CYP assay described here, reconstitute the lipid extract in 100 μl ethanol/water (70/30; v/v). *Note. A small amount of water is added to the sample solvent to reduce column sensitivity to ethanol, which can have an effect on peak resolution. For example, injection of 100% ethanol on the Kinetex C18 column will produce broad, split peaks at the start of the assay.* However, when planning to use the chiral assay, you should reconstitute the samples in 100 μl ethanol. *Note. Using an aqueous solution with the Cellulose-1 column used for the chiral assay can result in longer analyte retention times.* Transfer the lipid extract using a gas-tight glass syringe into a clean glass insert within an amber glass vial and tightly seal. The lipid extracts can be stored frozen at −20 °C for 1 week although it is advisable to run them as soon as possible after extraction.

### Preparation of lipid mediator standards

*Note. All lipid solutions and extracts should be prepared and handled using fixed-needle gas-tight glass syringes.* Prepare working standard solutions of 10 ng/μl in ethanol, for every individual lipid standard. Mix appropriate volumes of each one of these working solutions to prepare 200 μl of a mixed standard solution at a final concentration of 400 pg/μl per sample, in ethanol. The composition of this mixed standard will depend on the assays planned. *Note. 15-Epi-LXA*_*4*_ *does not ionize as efficiently as LXA*_*4*_ *and as a result it gives a much lower signal during MRM analysis. Therefore, to have a good signal for 15-epi-LXA*_*4*_ *we found it necessary to use a higher concentration of this mediator in the mixed standard. This is particularly important when planning to compare the prevalence of 15-epi-LXA*_*4*_ *and LXA*_*4*_ *(e.g., chiral analysis). We are suggesting a final concentration of 4000* pg/*μl for 15-epi-LXA*_*4*_. For the internal standards, transfer 100 μl of PGB_2_-*d*_4_ (10 ng/μl) into a glass amber vial and add 900 μl of ethanol to prepare a 1-ml solution of 1 ng/μl. Repeat this step for 12(*S*)-HETE-*d*_8_ and prepare also a solution of 1 ng/μl.

To prepare solutions for calibration lines, transfer the appropriate volume (see below) of the mixed standard solution (400 pg/μl) into a clean glass insert vial. Add to it 40 ng of each internal standard (40 μl of 1 ng/μl solution of PGB_2_-*d*_4_ and/or 12(*S*)-HETE-*d*_8_) and evaporate the solvent to dryness under nitrogen. When dry, reconstitute the lipid residue with 100 μl of the appropriate solvent, as follows: use ethanol/water (70/30; v/v) when planning to run the COX or the LOX/CYP assay; use 100% ethanol when planning to run the chiral assay. Repeat this process using various volumes of the mixed calibration standards to achieve the following concentrations: (a) COX assay, 100, 50, 20, 10, and 2 pg/μl; (b) LOX/CYP assay, 100, 50, 20, 10, and 4 pg/μl; and (c) chiral assay, 160, 100, 50, 20, and 10 pg/μl.

### LC/ESI–MS/MS analysis of prostanoids

Optimal MS parameters should be determined for each individual lipid mediator by direct infusion and using a working standard solution of 10 ng/μl. MS analysis is conducted in electrospray negative mode. Typical MS/MS parameters for the Quattro Ultima are capillary voltage 4000 V, cone voltage 35 V, dwell time 0.2 s, source temperature 100 °C, desolvation temperature 400 °C, first and second analyzer (Q1 and Q2) resolution 14.0, and ion energy Q1 and Q2 1.5. The collision energy required for fragmentation of the individual lipid mediators needs to be experimentally established; the settings used for this protocol are shown in Table 1.

### LC/ESI–MS/MS for COX-derived mediators (COX assay)

The COX assay is designed to monitor lipid mediators that are generated from COX metabolism of DGLA, AA, and EPA and their 15-keto- and 13,14-dihydro-15-keto metabolites. A list of mediators included in this assay is shown in Table 1A.

1. Prepare the following: solvent A, acetonitrile/water/glacial acetic acid (45/55/0.02; v/v/v), and solvent B, acetonitrile/water/glacial acetic acid (90/10/0.02; v/v/v). These mobile-phase solvents can be kept at room temperature for 2 weeks.
2. Prepare a seal wash of acetonitrile/water (90/10; v/v) and a needle wash of acetonitrile/water (70/30; v/v). These solvents are required to prevent any cross-contamination of the HPLC and autosampler during the course of the assay. In addition, prepare the following column wash solvents: methanol/water/glacial acetic acid (50/48/2; v/v/v) and methanol/water (50/50; v/v). These solvents are to be used at the end of the experiment or in between batches of samples to clean the column.
3. Filter and degas all solvents before putting them onto the LC system. Purge the lines.
4. Connect the C18 Luna column and set the flow rate to 0.2 ml/min. Ensure that there are no leaks and wash the system with solvent A/solvent B (50/50; v/v) for 1 h.
5. Check for any background contamination on the MassLynx Tune page.
6. Set up the following gradient on the MassLynx inlet page: 0.0 to 8.0 min, 100% solvent A; 8.1 to 12.0 min, 50% solvent A; 12.1 to 20.0 min, 30% solvent A; 20.1 to 30.0 min, 100% solvent A.
7. Load inlet method and allow to equilibrate at 100% solvent A (initial gradient conditions) for 30 min. Set the sample chamber to 8 °C and injection volume at 10 μl. Check for any background contamination on the MassLynx Tune page.
8. Load the MassLynx MS method that has been programmed to scan in MRM mode for the analytes of interest.
9. Check for background contamination by setting up multiple blank injections (*n*=3) of ethanol/water (70/30; v/v) on the MassLynx sample list page.
10. Check instrument sensitivity and chromatographic resolution by setting 20 (midrange calibration) and 2 pg/μl (low-range calibration) standard injections (*n*=3) on the MassLynx sample list page.
11. If all instrument and chromatographic conditions are met begin sample analysis.
12. When the assay is complete, wash the column with methanol/water/glacial acetic acid (50/48/2; v/v/v) for 3 h followed by methanol/water (50/50; v/v) for a further 3 h. After the column has been washed, remove from the HPLC, replace end caps, and store.

### LC–MS/MS analysis of hydroxy and epoxy fatty acids (LOX/CYP assay)

The LOX and CYP assay is designed to monitor lipid mediators that are generated from the metabolism of LA, AA, EPA, and DHA by LOX or CYP enzymes. A list of mediators included in this assay is shown in Table 1B.

1. Prepare the following: solvent C, acetonitrile/water/glacial acetic acid (45/55/0.02; v/v/v), and solvent D, methanol/water/glacial acetic acid (80/20/0.02; v/v/v). The mobile-phase solvents can be kept at room temperature for 2 weeks.
2. Prepare seal wash, needle wash. and column wash solvents as described above (step 2, COX assay).
3. Filter and degas the solvents before putting them onto the LC system.
4. Connect the C18 Kinetex column and set the flow rate to 0.2 ml/min. Ensure that there are no leaks and equilibrate with solvent C/solvent D (50/50; v/v) for 1 h.
5. Check for any background contamination on the MassLynx Tune page.
6. Set up the following gradient on the MassLynx inlet page: 0.0 to 1.0 min, 70% solvent C; 1.1 to 25.0 min, 17% solvent C; 25.1 to 28.0 min, 0% solvent C; 28.1 to 35.0 min, 70% solvent C.
7. Load inlet method and allow to equilibrate at solvent C/solvent D (70/30; v/v) (initial gradient conditions) for 30 min. Set the sample chamber to 8 °C and injection volume at 10 μl. Check for any background contamination on the MassLynx Tune page.
8. Load the MassLynx MS method that has been programmed to scan in MRM mode for the analytes of interest.
9. Check for background contamination by setting up multiple blank injections (*n*=3) of ethanol/water (70/30; v/v) on the MassLynx sample list page.
10. Check instrument sensitivity and chromatographic resolution as described for the COX assay (step 10) and begin sample analysis.
11. When the assay is complete, wash and store the column as described for the COX assay, step 12.

### Chiral chromatography

**Fig. 4:**
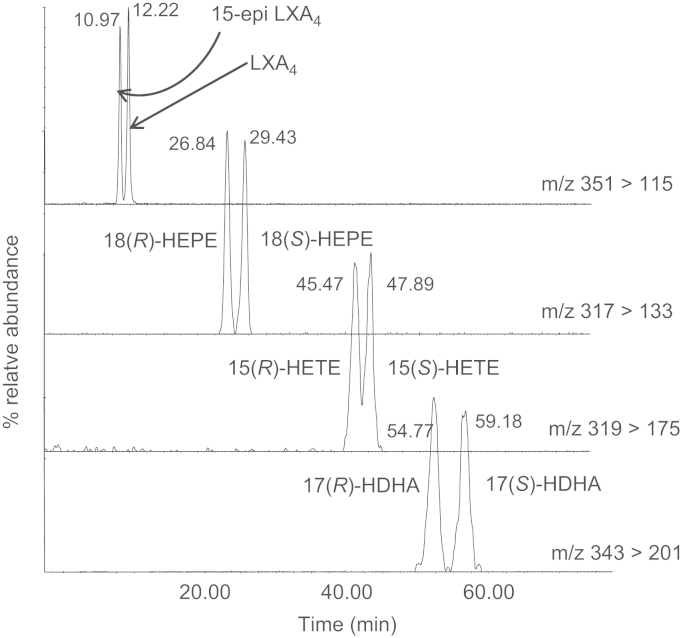
Typical chromatograms showing the chiral separation of the AA-derived 15-*epi*-LXA_4_ and LXA_4,_ EPA-derived 18(*R*)- and 18(*S*)-HEPE, the AA-derived 15(*R*)- and 15(*S*)-HETE, and DHA-derived 17(*R*)- and 17(*S*)-HDHA by LC/ESI–MS/MS (experimental conditions as described under Protocol; 15-*epi*-LXA_4_, 2.5 ng on the column; all other mediators, 250 pg on the column).

This protocol can be applied to various mono- and polyhydroxy fatty acids. Typical chromatograms showing the AA-derived ±12-HETE, ±15-HETE, LXA_4_ and 15-*epi*-LXA_4_, EPA-derived ±18-HEPE, and DHA-derived ±17-HDHA are presented in Fig. 4. *Note. The cellulose column used in this protocol has to be thoroughly conditioned with isopropanol before being used with aqueous mobile phases suitable for reverse phase-like separation.* A short list of some of the mediators that can be analyzed using this assay is shown in Table 1C.

1. Prepare the following solvents separately: (a) acetonitrile with 0.001% glacial acetic acid, (b) water with 0.001% glacial acetic acid, (c) methanol with 0.001% glacial acetic acid. *Note. It is necessary to prepare each solvent separately. The chiral column is very sensitive to any changes in mobile-phase composition and, to ensure reproducible retention times, is it advisable to mix the individual solvent components of the mobile phase via the inlet method of the HPLC.*
2. Prepare seal wash, needle wash, and column wash solvents as previously described (step 2, COX assay).
3. Filter and degas each solvent before putting it onto the LC system.
4. Connect the LUX Cellulose-1 column and set the flow rate to 0.2 ml/min. Ensure that there are no leaks and equilibrate with acetonitrile/water/methanol in 0.02% glacial acetic acid (39/58/3 v/v/v) for 3 h (see *Note* for step 1 above).
5. Load MassLynx Tune page and check for any background contamination.
6. Load inlet method and set the sample chamber to 8 °C and injection volume at 5 μl.
7. Load the MassLynx MS method that has been programmed to scan in MRM mode for the analytes of interest.
8. Check for background contamination by setting up multiple blank ethanol injections (*n*=3) on the MassLynx sample list page.
9. Check instrument sensitivity and chromatographic resolution as described for the COX assay (step 10) and begin sample analysis.
10. When the assay is complete, wash and store the column as described for COX assay (step 12).

### Protein content determination

For liquid samples remove a protein aliquot (typically 10–20 μl) before adding methanol to the sample. For solid samples either remove a small aliquot of the homogenate or collect the protein pellet after centrifuging the sample homogenate. Store the samples at −20 °C awaiting analysis. The protein pellet cannot be dissolved in water but it helps to suspend it in 1 M NaOH (1 ml) (1 M NaOH: 4 g NaOH in 100 ml water) and warm the solution in a water bath. The assay is carried out using a 96-well microplate format and following the manufacturer's instructions using BSA as the protein standard.

### Data analysis

Data analysis is carried out using MassLynx version 4.0. Analyte-related peaks are identified by the relevant MRM transition and retention time compared to the standards of the day. All peaks are integrated and the integrals are recorded in a spreadsheet. Analytes can be quantified with accuracy when the signal-to-noise (S/N) ratio is at least 10; this is defined as the limit of quantitation. When the S/N is at least 3, the analyte is at the limit of detection (LoD) of the method [53]. The S/N can be estimated using the operating software (MassLynx). The limit of detection of lipid mediators varies because of differences in ionization efficiency. For the assays reported here the LoD ranges as follows: 0.1 to 10 pg/μl for the COX assay, 0.1 to 20 pg/μl for the LOX/CYP assay, and 10 to 20 pg/μl for the chiral assay. For example, the LoD for PGE_2_ and 12-HETE in human plasma is 20 and 32 pg/ml, respectively, whereas when using the chiral assay, the LoD for 12(*S*)-HETE is 2 ng/ml. *Note. Using a chiral stationary phase has an additional impact on assay sensitivity due to unavoidable “column bleed.” This in turn produces an ion-suppression effect (see Caveats) with ESI that affects ionization efficiency. As a result the LoD for the chiral assay is higher (less sensitive) than that of assays using the more stable C18 stationary phase.*

## Calculations and expected results

Calibration lines are constructed using the least-squares linear regression method. To determine the concentration of any given analyte the peak area to internal standard ratio is calculated and read off the corresponding calibration line as concentration (pg/μl). The results are then normalized using the sample volume or the amount of protein or the wet tissue weight and reported as pg mediator/μl sample or pg lipid mediator/mg protein or pg lipid mediator/mg tissue, respectively. The assays reported here can be applied to biological, clinical, and in vitro samples.

### Example of results obtained from liquid biological samples

Cutaneous suction blister fluid samples (71±44 μl) were collected from healthy subjects (*n*=18) 12 h after blistering and after 28 day of supplementation with placebo (mineral oil; 2.4 ml) [44]. The concentrations of PGE_2_ and 12-HETE were estimated at 0.59±0.34 and 15.09±0.34 pg/mg protein, respectively. Analysis of plasma samples (799±145 μl) collected from the same individuals determined that the levels of PGE_2_ and 12-HETE were 0.28±0.11 and 0.05±0.03 pg/μl, respectively. In addition, chiral analysis of the lipid extract obtained from the cutaneous blister fluid identified 12(*S*)-HETE as the dominant enantiomer, suggesting that this was not a product of oxidation but enzymatic oxygenation of AA through 12-LOX.

### Example of results obtained from solid biological samples

PGE_2_ and 12-HETE were identified in the pancreas of FAT-1 transgenic mice (*n*=4) at 9.4±3.82 and 49.5±30.2 pg/mg protein, respectively [39]. The CYP-derived mediator 11(12)-EET has been identified in benign endometrial tumor at 165 pg/mg protein or 16.4 pg/mg tissue (unpublished data).

## Caveats

A limitation of ESI is the occurrence of ion-suppression (matrix) effects, which are typically caused by mobile-phase additives (such as acids and bases) and coeluting impurities from biological samples [54,55]. Such influences on analyte ionization efficiency can have an impact on instrument sensitivity. Sample related matrix effects can be minimized through sample cleanup by SPE. It is also advisable to use small injection volumes (typically 2–10 μl) and take appropriate measures during a sample run to keep the system as clean as possible, i.e., an appropriate needle wash or seal wash. Interspersing blank injections through the course of the run is a very useful way to monitor carryover and background contamination. Also the type and concentration of acid used in the mobile phase should be carefully selected.

Some other drawbacks may rise from the choice of internal standards used in the assays. Although ^13^C-labeled eicosanoids would have been a better choice, these are not commercially available, making deuterium-labeled standards the only accessible option. However, these standards do not have the exact same retention times as the corresponding unlabeled eicosanoids, and their presence may result in differential suppression or enhancement of ionization and, consequently, affect the quality of the analytical data [56,57]. The number of deuterium-labeled internal standards can vary according to the experimental design. Here, we have opted to use only two, namely PGB_2_-*d*_4_ and 12(*S*)-HETE-*d*_8_, as class-representative compounds (prostanoids and hydroxy fatty acids). Their recovery was comparable to that of the analytes of interest and in this way can compensate for any losses during extraction [33,34]. Whereas the ionization efficiencies of different lipid mediators vary and the two internal standards cannot account for all changes, quantitation is based on the use of calibration lines made with synthetic eicosanoid standards that ionize similar to the lipids of interest. Furthermore, the calibration lines are run on a daily basis, bracketing the biological samples and being spiked with the same internal standards used for the biological samples, addressing some of these concerns.

Systemic eicosanoid production, often used to evaluate various pathological conditions such as HIV, cancer, and renal disease, can be assessed through measurement of urinary metabolites (e.g., dinor- and tetranor-PGs) [58,59]. However, plasma eicosanoids are a better marker for blood cells and are used as inflammatory biomarkers often associated with the effectiveness of nutritional and other interventions [44,60]. Nevertheless, it should be noted that because of the rapid nature of eicosanoid metabolism, plasma mediators may not be fully representative of total in vivo production.

Finally, it should be pointed out that when collecting human or animal plasma samples, platelets may become activated and produce large quantities of 12-HETE. This problem can be minimized if blood is collected directly in containers with anticoagulant, but cannot always be avoided.

*Caution.* Keep all samples and standard solutions on ice throughout the experiment. Furthermore, lipids are prone to photodegradation; therefore avoid exposure to direct sunlight. Ethanol easily evaporates; therefore it is important to keep all sample extracts and solutions of standards in tightly sealed tinted vials. For long-term storage (stock solutions of standards) keep the solutions at −80 °C; for short-term storage (biological extracts or working solutions of standards) store the samples at −20 °C for no more than 1–2 weeks.

*Caution.* To avoid cross-contamination of lipid standards and biological extracts it is essential to rinse out all the glass syringes thoroughly using ethanol. It also advisable to use syringes dedicated to handling standards or biological sample extracts.

*Caution.* When using organic solvents in volumes greater than 10 ml appropriate safety measures should be taken. Methyl formate, methanol, and hexane are toxic.

*Caution.* There is a limit to the number of MRM transitions that can be recorded without compromising the sensitivity of the assay. Although this number is instrument specific, it is advisable to segment the MRM protocol in accordance with the retention time of mediators of interest.

## Figures and Tables

**Table 1:** Experimental conditions used for the LC/ESI–MS/MS analysis of polyunsaturated fatty acid-derived prostanoids, hydroxy fatty acids, and various chiral hydroxy fatty acid species.

Mediator	MRM (*m/z*)	CE (eV)	RT (min)
(A) Prostanoids			
15d-PGJ2	315→271	15	18.71
PGJ2	333→271	15	11.08
Δ12-PGJ2	333→271	15	11.99
15-Keto-PGE2	349→113	23	6.74
PGE3	349→269	15	4.03
PGD3	349→269	15	4.52
PGE2	351→271	17	4.85
PGD2	351→271	17	5.75
PGF2α	353→193	25	3.95
PGE1	353→317	15	4.93
PGD1	353→317	15	5.50
PGF1α	355→311	25	3.86
TXB3	367→169	15	3.04
TXB2	369→169	17	3.54
6-Keto-PGF1α	369→163	23	2.80
13,14-dh PGF2α	355→311	25	5.18
13,14-dh-15-k-PGE1	353→335	12	8.87
13,14-dh-15-k-PGE2	351→333	12	8.05
13,14-dh-15-k-PGF2α	353→113	28	6.90
13,14-dh-15-k-PGF1α	355→193	32	7.64
PGB2-*d*4	337→179	20	11.99

(B) Chiral hydroxy fatty acid species			
15-*Epi*-LXA_4_	351→115	20	10.97
LXA_4_	351→115	20	12.22
RvD_1_	375→141	15	13.43
RvE_1_	349→195	17	6.55
18(*R*)-HEPE	317→133	20	26.80
18(*S*)-HEPE	317→133	20	29.43
15(*R*)-HETE	319→175	18	45.45
15(*S*)-HETE	319→175	18	47.89
17(*R*)-HDHA	343→201	15	54.77
17(*S*)-HDHA	343→201	15	59.18
12(*R*)-HETE	319→179	17	51.68
12(*S*)-HETE	319→179	17	53.06
12(*S*)-HETE-*d*8	327→184	17	51.60

(C) Hydroxy fatty acids			
±9-HODE	295→171	25	15.00
±13-HODE	295→195	25	14.82
±5-HEPE	317→115	20	13.04
±8-HEPE	317→155	18	11.76
±9-HEPE	317→149	20	12.15
±11-HEPE	317→167	20	11.56
±12-HEPE	317→179	17	11.85
±15-HEPE	317→175	18	11.37
±18-HEPE	317→133	25	10.89
±5-HETE	319→115	20	19.38
±8-HETE	319→155	20	17.50
±9-HETE	319→123	20	19.02
±11-HETE	319→167	20	16.70
±12-HETE	319→179	17	17.33
±15-HETE	319→175	18	15.72
±10-HDHA	343→153	17	17.33
±13-HDHA	343→193	17	16.88
±14-HDHA	343→161	20	17.33
±17-HDHA	343→201	15	16.25
±20-HDHA	343→241	20	15.45
15(*S*)-HETrE	321→221	15	19.47
LTB_4_	335→195	17	8.39
LTB_5_	333→195	20	6.43
PD1	359→206	15	5.50
RvD_1_	375→141	15	4.16
RvE_1_	349→195	17	1.93
LXA_4_	351→115	20	4.10
5-*Oxo*-ETE	317→203	30	20.10
12(*S*)-HETE-*d*8	327→184	17	14.66
±5(6)-EET	319→191	20	23.58
±8(9)-EET	319→127	22	22.24
±11(12)-EET	319→208	15	21.44
±14(15)-EET	319→113	30	19.02
±5,6-DHET	337→145	25	13.39
±8,9-DHET	337→127	25	11.78
±11,12-DHET	337→167	22	11.03
±14,15-DHET	337→207	25	10.22

CE, collision energy; RT, indicative retention time.
